# Acute neurological signs as the predominant clinical manifestation in four dogs with *Angiostrongylus vasorum *infections in Denmark

**DOI:** 10.1186/1751-0147-53-43

**Published:** 2011-06-28

**Authors:** Hanne Gredal, Jakob L Willesen, Henrik E Jensen, Ole L Nielsen, Annemarie T Kristensen, Jørgen Koch, Rikke K Kirk, Susanne E Pors, Geoff C Skerritt, Mette Berendt

**Affiliations:** 1 Department of Small Animal Clinical Sciences, Faculty of Life Sciences, University of Copenhagen, Dyrlaegevej 16, DK-1870 Frederiksberg C, Denmark; 2 Department of Veterinary Disease Biology, Faculty of Life Sciences, University of Copenhagen, Ridebanevej 3, DK-1870 Frederiksberg C, Denmark; 3 Novo Nordisk A/S, Novo Nordisk Park, DK-2760 Måløv, Denmark; 4 ChesterGates Referral Hospital, Telford Court, ChesterGates, Chester CH1 6LT, UK

## Abstract

Four dogs with acute neurological signs caused by haemorrhages in the central nervous system were diagnosed with *Angiostrongylus vasorum *infection as the underlying aetiology. Two dogs presented with brain lesions, one dog with spinal cord lesions and one with lesions in both the brain and spinal cord. Only one dog presented with concurrent signs of classical pulmonary angiostrongylosis (respiratory distress, cough), and only two dogs displayed overt clinical signs of haemorrhages. Results of coagulation assays were inconsistent. Neurological signs reflected the site of pathology and included seizures, various cranial nerve deficits, vestibular signs, proprioceptive deficits, ataxia and paraplegia. One dog died and three were euthanised due to lack of improvement despite medical treatment. This emphasises canine angiostrongylosis as a potential cause of fatal lesions of the central nervous system and the importance of including *A. vasorum *as a differential diagnosis in young dogs with acute neurological signs in Denmark.

## Background

*Angiostrongylus vasorum*, also known as the French heartworm, is an approximately 13-21 millimetre gastropod-borne nematode, recognised in endemic areas of Europe [1-8], North and South America [9-12] and Uganda [13,14]. In endemic areas, the parasite is a well-known cause of respiratory disease (canine pulmonary angiostrongylosis, CPA) especially in young dogs which, along with other canine species, e.g. the fox, act as the definitive hosts in the life cycle of the parasite [1,3,15]. The adult worms primarily inhabit the pulmonary arteries and right cardiac ventricle of the canine host, resulting in a verminous pneumonia with obliterative thrombotic endarteritis and fibrosis [16]. The primary clinical signs are reported to be cough, exercise intolerance, dyspnoea and right sided heart failure, all of which are directly related to the respiratory system. However, non-specific clinical signs such as vomiting, diarrhoea or anorexia are also frequently reported [3]. Furthermore, disorders of haemostasis resulting in severe haemorrhages have been identified as possible complications of the CPA complex [3,15]. The exact pathophysiological mechanisms remain unclear [3,15], but a chronic low grade disseminated intravascular coagulation (DIC) with associated consumption of both platelets and coagulation factors is most commonly suggested [17,18]. Immunemediated thrombocytopenia associated with *A. vasorum *infections has also been reported as a possible cause of bleedings [19]. Prolongation of clotting times and decrease of coagulation factors, e.g. von Willebrand factor and factor V have been reported in both experimentally and naturally infected animals, although not consistently [17,20,21].

A minor proportion (approximately 4%) of CPA patients present with neurological signs [3,15]. Acute neurological signs including ataxia, paresis, paralysis, or seizures seem to result primarily from haemorrhages in the central nervous system (CNS) caused by the parasitic induced haemostatic disorder [2,22-25]. This has previously been reported from the United Kingdom [20,22,23,26] and Germany [2]. However, migrating larvae, causing focal damage to the nervous tissue have also been reported [25,27]. A recent case report suggests that *A. vasorum *may cause an inflammatory response of the CNS resembling that of a meningitis or meningoencephalitis [28].

Previous investigations of populations of clinically affected CPA patients, including dogs with neurological signs, report a median age of 10 to 18 months [15,29]. Neurological signs in young dogs often give rise to a suspicion of congenital/hereditary conditions or immune-mediated meningitis/meningo-encephalitis, whereas CPA may easily be overlooked as a differential diagnosis, potentially resulting in a fatal outcome in these young dogs.

Neurological cases of dogs with *A. vasorum *have not previously been reported from Denmark. The purpose of the present study was therefore to draw attention to *A. vasorum *infections as a potential cause of acute neurological disease in dogs in Denmark. We describe the diagnostic work-up, medical treatment and outcome in four dogs, presenting with neurological signs caused by CNS haemorrhages associated with haemostatic dysfunction in dogs infected with *A. vasorum*.

## Case presentations

All cases were seen at the small animal hospital, University of Copenhagen, Denmark. A summary of the clinical data and blood results can be appreciated from Additional file 1: Table S1 and Additional file 2: Table S2.

### Case 1

A 31/2-year-old male Welsh Corgi was presented as an emergency with severe ataxia of acute onset a few hours prior to admission. A clinical examination revealed a bleeding wound in the tongue. The dog had previously been investigated for haemostatic dysfunction due to recurring episodes of prolonged bleeding and subcutaneous haemorrhages within six months of the acute neurological event. An underlying cause was not identified at the time and no medical treatment was given.

The dog presented non-ambulatory, mentally depressed and with tonic-clonic seizures. There was spontaneous nystagmus, changing between rotatory and vertical, and all limbs were slightly hypertonic. Postural reactions were not tested as the dog was non-ambulatory. These findings indicated one or more neurological lesions located to the vestibular system (central or peripheral) and/or the cerebellum in addition to cerebrocortical involvement.

Routine biochemistry revealed hypercalcaemia and hyperglycaemia, but other results were within normal references (additional file 2). Regrettably, haematology and coagulation times were not performed at this point in time. Previous laboratory results had revealed normal platelet counts and marginally elevated coagulation times in addition to increased D-dimers (8.7) (additional file 2).

Due to a history of recurrent signs of haemostatic dysfunction, a brain haemorrhage was considered the most likely cause of acute neurological disease, although the underlying bleeding disorder had not been characterised. However, neuroimaging was not available at the hospital for further diagnostic work-up.

The dog was hospitalised for supportive care (intravenous (IV) fluids and oxygen) and anticonvulsant therapy (diazepam at 1 mg/kg IV, phenobarbitone at 3 mg/kg with a subsequent administration 1 h later). Unfortunately, the dog deteriorated continuously and was euthanised on the same day at the owner's request.

Necropsy and histopathology of the brain (haematoxylin and eosin (H&E)) demonstrated the presence of acute disseminated haemorrhages throughout the brain and meninges, with larger solitary bleedings located to the right part of the cerebellum, and to the left caudate nucleus and the internal capsule. Furthermore, acute malacic foci and foci of gitter and glial cells, oedema and proliferation of capillaries/venules were demonstrated throughout the brain, including the cerebral cortex. Moderate cuffing and meningeal infiltrates of macrophages and lymphocytes/plasma cells and neutrophils were also present. Additionally, a larval granuloma was recorded in the encephalon.

In the lungs, multiple adult *A. vasorum *worms and thrombosis of pulmonary arteries with proliferation of the intima layer were acknowledged. Interstitial pulmonary fibrosis and granulomas with infiltrating macrophages as the predominant cell type and a central content of eggs or larvae were found throughout the lung tissue.

### Case 2

An eleven-months-old male Basset Hound was referred as an emergency with a two-week-history of respiratory signs, including tachypnoe and coughs, and an acute onset of tonic-clonic seizures with reduced consciousness 24 h prior to presentation.

On presentation the dog was mentally depressed and non-ambulatory tetraparetic and showed severe respiratory signs. Neurological signs progressed rapidly and a few hours after presentation the dog was semi-comatous with absence of pupillary light reflexes (PLR) and anisocoria. Given a history of seizures and rapidly progressing mental changes, cerebrocortical pathology causing increased intracranial pressure and possible brain stem affection, was suspected.

Thoracic radiographs displayed signs of focal lung pathology in the caudal lung lobes which could indicate *A. vasorum *infection. A diagnosis was verified by a faeces smear containing multiple L1 larvae. Routine haematology revealed a reduced packed cell volume (PCV) of 0.33 and a platelet count of 97 × 10^9^/L. Activated partial thromboplastin time (APTT) was normal and prothrombin time (PT) and D-dimer were only slightly elevated (additional file 2). Given the diagnosis of severe pulmonary angiostrongylosis and thrombocytopenia, a CNS haemorrhage was considered likely, although no overt bleedings were present. Intoxication was also considered, but was highly unlikely according to the owner. Intensive supportive medical care and imidacloprid 10%/moxidectin 2.5% (Advocate spot-on) at 0.1 ml/kg were instituted, but the dog deteriorated and died within hours of admission to the hospital.

**Figure 1:**
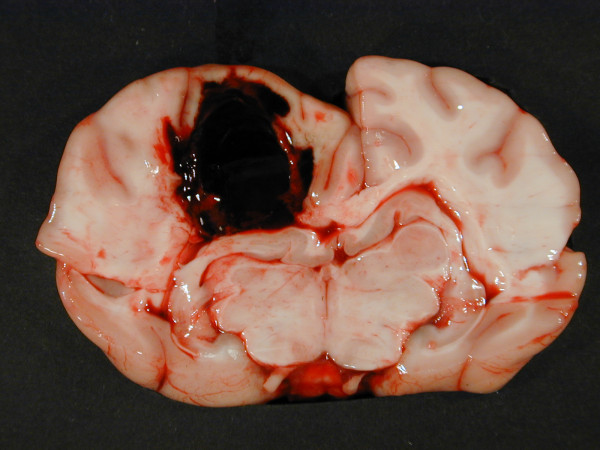
**Transverse section cerebrum at the level of hippocampus, case 2**. A haematoma, 2.5 centimetre in diameter, is seen in the left cerebral hemisphere compressing the surrounding brain tissue.

**Figure 2:**
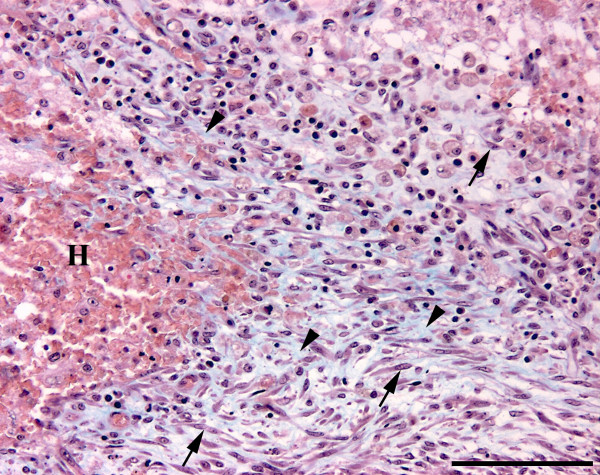
**Histological section of the brain, case 2**. Haematoma (H) with peripheral fibroplasia indicated by the proliferation of fusiforme cells (arrows) and the generation of collagen (blue) (arrow heads). Masson trichrome. Bar = 100 μm.

**Figure 3:**
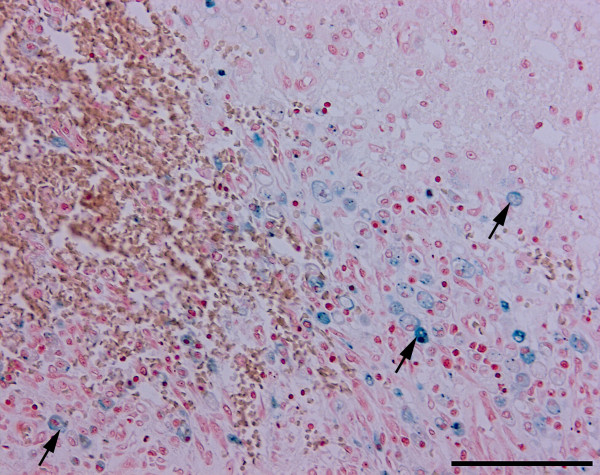
**Histological section of the brain, case 2**. Gitter cells (macrophages) (arrows) containing haemosiderin (blue). Perl's Prussian Blue stain. Bar = 100 μm.

At necropsy, a subcutaneous haematoma of 10 × 20 centimetres was detected on the left side of the thorax. In the left cerebral hemisphere, a haematoma, 2.5 centimetres in diameter, was extending into the left lateral ventricle (Figure 1). Histopathology (H&E) of the area peripheral to the cerebral haematoma revealed the presence of gitter cells and changes compatible with granulation tissue including proliferated fusiforme cells, and capillaries/venules and collagen as shown by Masson trichrome staining (Figure 2). In addition, several acute microscopic haemorrhages were observed in the brain. Acute malacic foci were also encountered as well as foci of gitter and glial cells. Gitter cells (macrophages) occasionally contained haemosiderin as identified by the Perl's Prussian Blue stain (Figure 3). Moderate cuffing and meningeal infiltrates of macrophages, lymphocytes/plasma cells and neutrophils were identified in addition to dense focal infiltrates of neutrophils (absence of eosinophilic granules by Luna staining).

**Figure 4:**
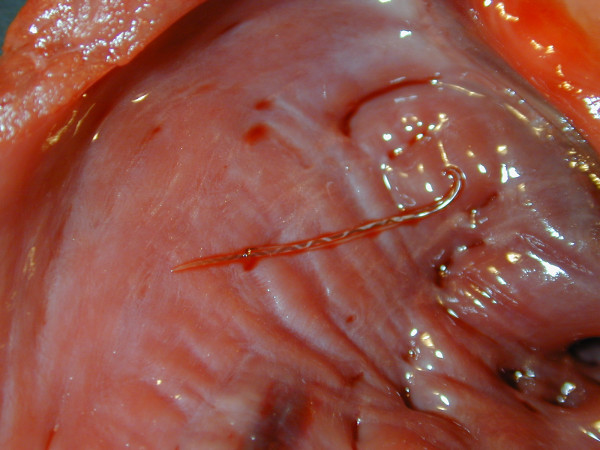
**The heart, case 2**. An adult Angiostrongylus vasorum is present in the right ventricle.

A single adult *A. vasorum *was present in the right cardiac ventricle (Figure 4). Histopathological findings of the lungs were comparable to those described for case 1. However, in addition to chronic lung lesions, acute haemorrhages were observed.

In the kidneys, a single larval granuloma was identified.

### Case 3

A seven-months-old Danish/Swedish farmdog was referred with progressive ataxia and mucosal membrane petechiae of 24 h duration. A diagnosis of *A. vasorum *had already been established by a faeces smear performed by the local veterinarian 24 h prior to referral. On presentation, the dog was alert but non-ambulatory paraplegic and in pain. Petechial haemorrhages were present in both scleras, and oral and ocular mucosal membranes. The neurological examination revealed no cranial nerve deficits. Both thoracic limbs were hypertonic and there was a bilateral flaccid paralysis of the pelvic limbs. Proprioceptive placing reactions were normal in the thoracic limbs but absent in the pelvic limbs. Patellar and withdrawal reflexes were bilaterally absent in the pelvic limbs, but the perineal reflex was preserved. Cutaneous trunci reflex was normal. Neurological signs seemed compatible with Schiff-Sherrington syndrome, and a T3-L3 lesion was suspected. However, as the cutaneous trunci lesion was normal in the T3-L3 region, multifocal disease was considered with a possible lesion cranial to C6, giving rise to upper motor neuron signs of the thoracic limbs, and a second lesion in the lumbosacral plexus (L4-S2) causing lower motor neuron signs of the pelvic limbs.

Thoracic radiographs showed a diffuse interstitial lung pattern. Haematology revealed a decrease in PCV (0.34), thrombocytopenia (103 × 10^9^/L), and increased D-dimers (2.0). Prothrombin time was prolonged (9.1) but APTT was only mildly increased (10.9) (additional file 2). The clinical findings of scleral and mucosal membrane haemorrhages strongly suggested the neurological signs to be caused by acute CNS haemorrhages. Based on previous experiences, *A. vasorum *was considered a likely cause of haemostatic dysfunction although other disorders such as von Willebrand deficiency and acute warfarin poisoning were also considered. The dog showed no improvement despite intensive treatment with IV fluids, plasma transfusion, fenbendazole (at 20 mg/kg once a day (SID)), methadone and prednisolone acetate IV (at 1 mg/kg SID). It was therefore euthanised at the owners' request after two days of hospitalisation.

**Figure 5:**
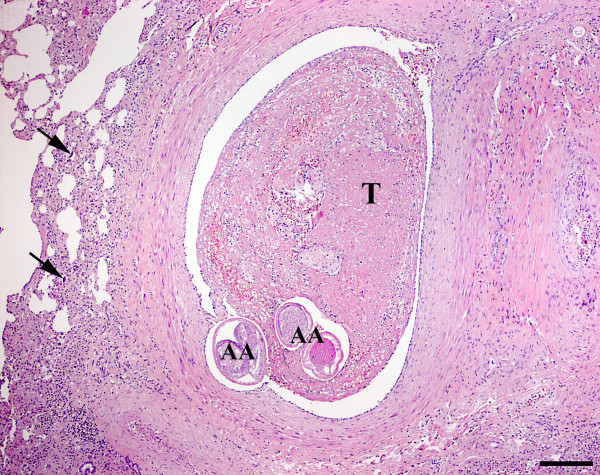
**Histological section of the lungs, case 3**. Thrombosis (T) of a pulmonary artery containing adult *Angiostrongylus vasorum *(AA). Haematoxylin and eosin. Bar = 200 μm.

At necropsy a haematoma was identified intramedullary in the caudal part of the spinal cord. Histopathology (H&E) of CNS furthermore revealed the presence of multiple acute focal haemorrhages primarily located to the left cerebral hemisphere. Occasionally, acute malacic foci were encountered, as well as foci of gitter and glial cells, oedema and proliferation of capillaries/venules. Moderate cuffing and meningeal infiltrates of macrophages, lymphocytes/plasma cells and neutrophils were also present. Histopathological findings of the lungs were comparable to those of case 1 (Figure 5).

### Case 4

A 10-month-old Labrador-Cross, female, was referred for investigation of rapidly progressing pelvic limb paresis and lumbosacral pain of four days duration.

**Figure 6:**
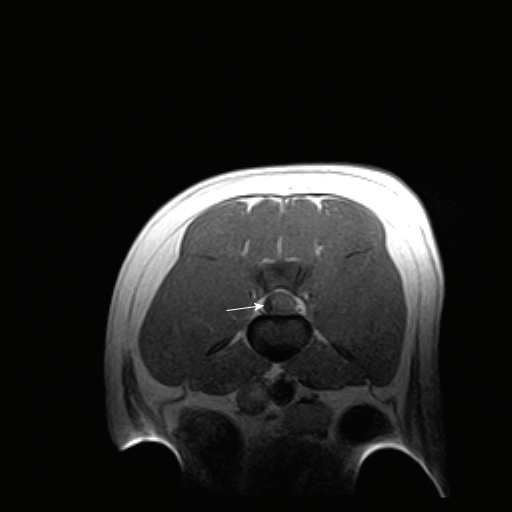
**Magnetic resonance imaging of the lumbar spinal cord, case 4**. Transverse T1 weighted images of the spinal cord at the level of vertebrae L5 revealing a hypointense area (arrow) lateralised to the right and compressing the spinal cord towards the left.

**Figure 7:**
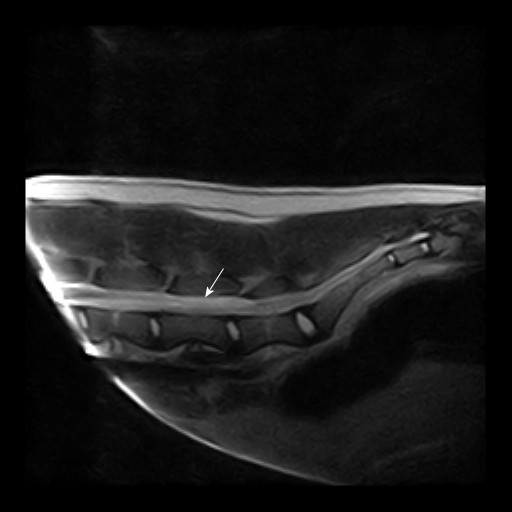
**Magnetic resonance imaging of the lumbosacral area, case 4**. Sagital T2 weighted images of the lumbosacral spinal cord demonstrating a hyperintense area (arrow) compatible with oedema caudal to the suspected haemorrhage.

On neurological examination immediately upon admission, the dog was alert, but unable to bear weight on its pelvic limbs. There were no cranial nerve deficits and thoracic limb reflexes and reactions were normal. Proprioceptive placing reactions were bilaterally absent in the pelvic limbs. Patellar reflexes were absent and withdrawal reflexes were reduced, whereas the perineal reflex was normal. The cutaneous trunci reflex was slightly decreased in the lumbar region. Deep pain perception and some voluntary movement were initially preserved, but the dog's neurological status gradually worsened during hospitalisation with complete loss of withdrawal reflexes and voluntary movement of the pelvic limbs. Based on the neurological findings, a spinal cord lesion located at segments L4-S2 was suspected. Thoracic radiographs revealed a diffuse lung pattern of interstitial and peribronchial hyperdensity. Blood analysis revealed a slightly decreased PCV (0.35), mild thrombocytopenia (145 × 10^9^/L), hyperglobulinaemia (72) and slightly increased D-dimers (0.6 mg/L) (Additional file 2: Table S2). A magnetic resonance imaging scan (Esaote vetscan 0.2 tesla) with T1- and T2-weighted images (WI) of the lumbosacral region was performed in transverse and sagital planes. Contrast was not administered. On T1 weighted images (T1-WI), a hypointense area was recognised subdurally at the level of vertebrae L4-L6, lateralised to the right and compressing the spinal cord towards the left (Figure 6). The same finding appeared iso- to hyperintense on T2 weighted images (T2-WI). Caudal to this was an area of hyperintense swelling on T2 (Figure 7). These findings were considered compatible with an acute haematoma with peripheral oedema in the area. A Baermann test was accordingly performed revealing multiple *A. vasorum larvae*.

Surgical spinal cord decompression at L4-L6 was considered, but did not seem a reasonable option given the risk of severe haemorrhages. The dog was therefore treated medically with plasma transfusions, fenbendazole at 20 mg/kg SID, prednisolone at 1 mg/kg SID for 3 days, methadone and physiotherapy for 6 days. However, as no improvement of the neurological status was recognised, the dog was euthanised at the owner's request.

At necropsy, an acute intramedullary haemorrhage was identified at the level of the caudal lumbar segments. In addition, several microscopic haemorrhages in the spinal cord were identified by histopathology (H&E). Occasionally, acute malacic foci were seen, as well as foci of gitter and glial cells, and proliferation of capillaries/venules. Moderate cuffing (macrophages, lymphocytes/plasma cells and neutrophils) and dense focal infiltrates of neutrophils (absence of eosinophilic granules by Luna staining) were furthermore encountered. Histopathological findings of the lungs were comparable to those of the previous cases. In the kidney a larva without any inflammatory reaction was seen within a small size vessel.

## Discussion

CPA is a commonly diagnosed disease in endemic areas of Denmark and the UK, primarily presenting as respiratory disease [3,15]. The included cases of the present study all presented with neurological signs as the primary complaint, in each patient caused by one or more CNS haemorrhages resulting from haemostatic dysfunction associated with *A. vasorum *and/or evidence of aberrant larvae. This emphasises CPA as an important differential diagnosis in dogs presenting with neurological signs of unknown aetiology. Acute non-traumatic brain haemorrhages are only infrequently reported in dogs [30], and *A. vasorum *should therefore be highly suspected as the underlying cause of brain haemorrhages in endemic areas. As opposed to ischaemic stroke, which appear with greater frequency and which might present with similar neurological signs, CPA is most commonly seen in young dogs [3,15], whereas the mean age of dogs reported with ischaemic stroke is 8.4-8.9 years [31,32]. The mean age of dogs in the present study was 10 months (median; 17 months) which is in accordance with previous studies of dogs naturally infected with *A. vasorum *[3,15]. The present study furthermore confirms haemorrhages due to *A. vasorum *to be an important differential diagnosis to acute disc disease and fibrocartilaginous embolisation in dogs with acute spinal cord disease in endemic areas as previously reported [23,33].

On clinical examination, only one dog (case 2) exhibited respiratory signs as part of the clinical pattern, and only two dogs (cases 1 and 3) had visible signs of an ongoing bleeding disorder. One of these dogs (case 1) had primarily haematomas, a characteristic clinical sign observed in connection with coagulopathies, whereas one dog (case 3) presented with mucosal petecchiation, a sign most often associated with disorders of primary haemostasis such as thrombocytopenia. Thus, CPA may easily be missed as the underlying aetiology due to the lack of classic respiratory signs or overt clinical signs of haemorrhages in dogs with acute neurological disease.

As opposed to dogs mildly to moderately infected (i.e. with no signs of coagulopathy or neurological disease), in which survival is close to 100% [3,15], the prognosis of CPA should be considered guarded when complicated by haemostatic dysfunction and even more so when the condition gives rise to severe neurological deficits. This can also be appreciated from a review of neurological cases in which 11 out of 17 (65%) died despite recognition and treatment of *A. vasorum *in the majority [2,20,22,23,25,26,28,33,34]. Compromised blood supply to the area involved, along with direct physical compression caused by a haematoma makes the CNS, which is strongly dependent on continuous supplies of oxygen and glucose, highly susceptible to irreversible damage. Furthermore, any attempt of surgical decompression or evacuation of the haematoma is severely complicated by the presence of ongoing haemostatic dysfunction unless addressed appropriately by blood component therapy such as fresh frozen plasma or cryoprecipitate [35].

In the present report all patients suffered from haemostatic dysfunction related to the parasitic infection according to the laboratory analysis, and all had a fatal outcome. Case 1 was seen back in 2000 when CPA was largely unknown in Denmark. Retrospectively viewed, CPA should have been considered the underlying aetiology of the coagulopathy when the dog first presented with subcutaneous haemorrhages only. Chances of a successful treatment at this early stage had probably been favourable.

In case 2 the dog deteriorated rapidly upon admittance despite appropriate diagnostic work-up and treatment. With thrombocytopenia, a low fibrinogen, a prolonged APTT and increased D-dimers the dog appeared to suffer a state of irreversible DIC resulting in a severe consumptive coagulopathy which was refractory to medical treatment, stressing the severe complications of *A. vasorum *infections [36]. Also notable in this case were the chronic histopathological changes indicating that the cerebral haematoma had been present for 7-10 days although acute neurological signs were not noticed until the day prior to submission. This emphasises the importance of early recognition and treatment of *A. vasorum *in dogs displaying other signs of CPA e.g. respiratory disease as in the present case.

In cases 3 and 4 the neurological condition caused by CPA did not appear to be fatal, but the dogs were euthanised as the prognosis with regard to full recovery and ambulation was guarded in both. Thus, death may not result directly from the haemorrhage itself, but euthanasia may be reasoned by the severe and irreversible disabilities caused by the haemorrhage.

On haematology, cases 2-4 presented with anaemia and thrombocytopenia. These results differ from findings in most mildly to moderately affected CPA patients [15,37] but have been reported in several neurological patients with haemostatic dysfunction [2,22] and in the study of experimentally infected dogs by Cury and others [17]. Regrettably, haematology and coagulation times were not performed at the final consult of case 1.

Eosinophilic counts were within normal ranges for all cases as opposed to the findings by Cury and others [17] and Chapman and others [15]. Although an eosinophilic response is to be expected with parasitic diseases [38], some studies report eosinophilia as a less frequent finding in *A. vasorum cases*. In this respect, Willesen and others found that only 21% of the dogs had eosinophilia at the time of diagnosis [37]. In addition, histopathology of the present cases demonstrated the absence of eosinophilia which is in accordance with previous reports of absent or only mild eosinophilia [2,27]. The exact mechanisms for this lack of eosinophilic response remain unclear, but have been suggested to be associated with either low grade infections or more chronic states [39,40].

D-dimers were analysed in cases 2-4 when they presented as neurological emergencies, and had previously been tested in case 1, and found elevated in all cases. A normal D-dimer level is known to be a valuable marker when ruling out deep venous thrombosis, pulmonary thromboembolism and DIC [36,41]. The findings of elevated D-dimers imply that DIC cannot be excluded as part of the underlying mechanism of *A. vasorum *induced coagulopathies.

Biochemistry revealed elevated levels of bilirubin in three cases, but otherwise normal results. Hyperbilirubinaemia, in the absence of elevated liver enzymes, is possibly the result of increased haemolysis in these suspected DIC patients. Previous reports of decreased levels of serum fructosamine and increased levels of serum globulin in CPA were generally not confirmed in this study [15,37,42]. Hyperglobulinaemia was only present in one case (case 4) (25%) as opposed to the findings by Chapman and others in which 70% had elevated serum globulins [15]. Hypercalcaemia was found in cases 1 and 3. This finding has previously been reported in association with *Angiostrongylus *infections in dogs and has been suggested to be the result of granulomatous disease [43]. As all dogs in the present study died, changes in calcium levels with antiparasitic treatment could not be followed.

## Conclusions

This report of four dogs infected with *A. vasorum *documents the importance of *A. vasorum *infection as a differential diagnosis, particularly in young dogs with acute neurological signs of unknown aetiology in Denmark. The identification of *A. vasorum *as the underlying cause of a primary presentation of neurological disease is complicated by the fact that dogs may display neither visible clinical signs of haemostatic dysfunction nor the classical respiratory signs of CPA.

We suggest that a Baermann test is performed in any dog with unexplainable neurological signs in endemic areas. Due to the intermittent shedding of *A. vasorum *larvae we advice testing of faeces samples from three consecutive days in order to increase the sensitivity of the test.

## Consent

Written informed consent was obtained from the owners for publication of this case report.

## Competing interests

The authors declare that they have no competing interests.

## Authors' contributions

HG headed the study, coordinated the collection of data of the four cases and was the main responsible for drafting the manuscript. JLW contributed with substantial information on all cases as well as participating in the design of the study and writing of introduction and discussion. ATK contributed with ideas for the study design and substantial information on haemostatic dysfunction in dogs with *A. vasorum*. JK contributed with data on all cases. HEJ, OLN, RKK and SEP performed post mortem examinations and histopathological investigations of all cases and drafted this part of the manuscript. GCS helped to draft the manuscript and contributed with background information on Angiostrongylosis in the UK. MB conducted the overall study, contributed with data on cases 1-4 and helped to draft the manuscript.

All authors read and approved the final manuscript.

## Supplementary Material

Additional file 1 **Table S1 A summary of signalment and clinical signs in 4 dogs with CNS haemorrhages associated with *A. vasorum***. Click here for file

Additional file 2 **Table S2 Selected blood results in 4 dogs diagnosed with *A. vasorum***. PCV packed cell volume, PT prothrombin time, APTT activated partial thromboplastin time (individual references in brackets). Pool = internal reference value Click here for file
