# Supplementary material for: Acute neurological signs as the predominant clinical manifestation in four dogs with Angiostrongylus vasorum infections in Denmark
Source: Acta Vet Scand. 2011 Jun 28;53(1):43. doi: 10.1186/1751-0147-53-43 (PMC3141560; doi:10.1186/1751-0147-53-43)
Supplement: Additional file 1 — Table S1 A summary of signalment and clinical signs in 4 dogs with CNS haemorrhages associated with A. vasorum. [file 1751-0147-53-43-S1.DOC]

| **Case** | **1** | **2** | **3** | **4** |
| --- | --- | --- | --- | --- |
| **Breed** | Welsh corgi | Basset | Danish Swedish farmdog | Labrador cross |
| **Gender** | Male | Male | Male | Female |
| **Age in months** | 42 | 11 | 7 | 10 |
| **Respiratory signs** | ÷ | + | ÷ | ÷ |
| **Clinically overt haemorrhages** | + | ÷ | + | ÷ |
| **MRI available** | ÷ | ÷ | ÷ | + |
| **Histopathology available** | Lungs, brain | Lungs, brain, kidneys | Lungs, brain, spinal cord | Lungs, spinal cord, kidneys |
| **Outcome** | Euthanasia | Death | Euthanasia | Euthanasia |
