# Supplementary material for: Acute neurological signs as the predominant clinical manifestation in four dogs with Angiostrongylus vasorum infections in Denmark
Source: Acta Vet Scand. 2011 Jun 28;53(1):43. doi: 10.1186/1751-0147-53-43 (PMC3141560; doi:10.1186/1751-0147-53-43)
Supplement: Additional file 2 — Table S2 Selected blood results in 4 dogs diagnosed with A. vasorum. PCV packed cell volume, PT prothrombin time, APTT activated partial thromboplastin time (individual references in brackets). Pool = internal reference value [file 1751-0147-53-43-S2.DOC]

**Table 2** Selected blood results in 4 dogs diagnosed with *A. vasorum*.

PCV packed cell volume, PT prothrombin time, APTT activated partial thromboplastin time (individual references in brackets). Pool= reference from control dogs

| **Case no.** | **1**  6 months prior to neuro. disease | **1**  4 months prior to neuro. disease | **1**  Final visit | **2** | **3** | **4** | **Reference range** |
| --- | --- | --- | --- | --- | --- | --- | --- |
| **Haematology** |  |  |  |  |  |  |  |
| Leucocytes total | 18.6 | 26.5 | - | 10.7 | 27.9 | 11.7 | 6.5-18.10x10^9^/L |
| Lymphocytes | 4.46 | 3.98 | - |  | 2.11 | 3.55 | 1.00-4.80x10^9^/L |
| Monocytes | 1.48 | 2.92 | - | 1.71 | 1.41 | 0.51 | 0-1.2x10^9^/L |
| Eosinophils | 0 | 0.8 | - | 0 | 0 | 0.52 | 0-1.2x10^9^/L |
| PCV | 0.43 | 0.28 | - | 0.33 | 0.34 | 0.35 | 0.39-0.59 |
| Platelets | 324 | 215 | - | 97 | 103 | 145 | 200-500x10^9^/L |
| **Coagulation** |  |  |  |  |  |  |  |
| Fibrinogen | - | - |  | 0.82 | 2.09 | 0.8 | 1.00-4.00 g/L |
| PT | 10  (6.5-9.5) | 9.8  (6.5-9.5) |  | 9.5  (pool; 7.6) | 9.1  (pool; 6.5) | 7.9  (pool; 7.6) | - |
| APTT | 18.7  (17.5-26.3) | 27.8  (17.5-26.3) |  | 10.7 seconds (pool; 12.4) | 10.9  (pool; 10.4) | 11.2  (pool; 10.4) | - |
| D-dimer | 8.7  (0-0.5) | 1.2  (0-0.5) |  | 1.3  (pool; 0.4) | 2.0  (pool; 0.4) | 0.6  (pool; 0.3) | 0.0-0.5 mg/L |
| **Biochemistry** | - | - |  |  |  |  |  |
| Glucose | - | - | 7.7 | 7.5 | 7.2 | 6.9 | 3.90-6.6 mmol/L |
| Bilirubin total | - | - | 13 | 7 | 7 | 1 | 0-5 μmol/L |
| Albumin | - | - | 38 | 40 | 35 | 32 | 26-44 g/L |
| Globulin | - | - | 43 | 53 | 44 | 72 | 23-52 g/L |
| Total protein | - | - | 80 | 93 | 79 | 103 | 57-82 g/L |
| Fructosamine | - | - | - | 387 | 265 | 248 | 235-344 μmol/L |
| Calcium |  |  | 3.78 | 3.10 | 3.8 | 2.51 | 2.2-3.3 mmol/L |
